# Priority areas and implementation of ecological corridor through forest restoration to safeguard biodiversity

**DOI:** 10.1038/s41598-024-81483-y

**Published:** 2024-12-28

**Authors:** Mayara Guimarães Beltrão, Camila Francisco Gonçalves, Pedro H. S. Brancalion, Ana Paula Carmignotto, Luis Fábio Silveira, Pedro Manoel Galetti, Mauro Galetti

**Affiliations:** 1 Museu de Zoologia da Universidade de São Paulo, São Paulo, SP Brazil; 2 Programa de Pós-Graduação em Ecologia e Recursos Naturais, Universidade Federal de São Carlos, São Carlos, SP Brazil; 3 Departamento de Ciências Florestais, Escola Superior de Agricultura Luiz de Queiroz, Piracicaba, SP Brazil; 4 Departamento de Biologia, Universidade Federal de São Carlos, Sorocaba, SP Brazil; 5 Departamento de Genética e Evolução, Universidade Federal de São Carlos, São Carlos, SP Brazil; 6 Departamento de Biodiversidade, Universidade Estadual Paulista, Rio Claro, SP Brazil; 7 Centro de Pesquisa em Biodiversidade e Mudanças do Clima, Instituto de Biociências, Universidade Estadual Paulista, Rio Claro, SP Brazil

**Keywords:** Functional connectivity, Enhanced Vegetation Index, Forest cover, Forest restoration, Atlantic Forest, Pernambuco Endemism Center, Ecology, Conservation biology, Restoration ecology

## Abstract

Ecological Corridors (ECs) are proposed as cost-effective solutions to improve ecological connectivity in fragmented landscapes. Planning the implementation of ECs must take into account landscape features as they affect the viability of the endeavor and the ECs associated costs. A novel set of geoprocessing tools were used to assess (i) economic viability; (ii) socioeconomic cost-effectiveness; and (iii) to determine priority targets for ECs establishment in a highly fragmented region of Atlantic Forest. Landscape features (e.g. size, edge density, number of patches), land use and cover, and Enhanced Vegetation Index data were obtained from remote sensing. Composition and configuration landscape metrics were estimated by geostatistical methods. Study area was represented by 35,344 forest fragments, of which 94% were composed of fragments smaller than 10 hectares in size, leading to a landscape characterized by low connectivity. We identified 13 priority fragments for protection and propose five ecological corridors. These five ECs connect six priority fragments, with a total of 283.93 ha of area to restore, on an estimated cost of nearly US$550.000,00. Conserving the biodiversity of one of the most threatened and fragmented tropical rainforest regions could be promoted at modest costs, through the protection of key fragments and their reconnection in the landscape.

**Supplementary Information:**

The online version contains supplementary material available at 10.1038/s41598-024-81483-y.

## Introduction

Human population growth increases the demand for food production, which drives agricultural expansion. This results in habitat loss and fragmentation, posing a severe threat to biodiversity and ecosystem functioning^1^. Although some species may be resilient to habitat fragmentation due to their ecological characteristics and adaptability, others are more severely affected. The effects of fragmentation can vary widely among species, but in general it creates barriers to species dispersal, reduces gene flow and genetic diversity, and ultimately leads to local extinctions^2,3^.

In order to mitigate these losses, ecological corridors (ECs) have emerged as a solution to connect fragmented habitats and enhance regional natural networks^4^. ECs are defined as strips of native vegetation that connect isolated habitat fragments resulting from human activity. They facilitate individual dispersal, genetic flow, and the maintenance of ecosystem functions, thereby serving as a viable approach to conserving biodiversity by linking fragmented ecosystems^5^. These corridors are clearly defined geographical areas that are managed over time to ensure or restore ecological connectivity. They are often referred to by terms such as `linkages`, `safe passages`, or `permeability areas`^6^.

In formulating conservation decisions pertaining to the establishment of protected areas and the implementation of ecological corridors, it is imperative to consider the influence of landscape features on ecological processes and species distribution^7^. Factors such as landscape connectivity, forest cover, and vegetation health play a crucial role in regulating biological flow, including colonization and recolonization dynamics^8^. Additionally, aligning these decisions with the goals of the UN Decade on Ecosystem Restoration can further enhance the effectiveness of conservation strategies by promoting global efforts to restore and maintain critical ecological functions and connectivity^9^. Least-Cost Path analysis (LCP) is a useful tool to plan viable and cost-effective ECs, as it determines the optimal path connecting two patches within a cost surface, considering various criteria such as distance, and matrix resistance^10^. In recent years, ecosystem restoration has become a global priority for mitigating biodiversity loss and reversing degradation^11^. The strategic placement of evidence-based ECs that connect key forest fragments in landscapes can effectively increase connectivity and ensure the dispersal and gene flow of species.

In Brazil, a number of connectivity initiatives, including corridors of varying scales, have been proposed^12^. Nevertheless, there are still some crucial areas for biodiversity conservation that lack proposals based on scientific evidence. This study represents a data-driven scientific effort aimed at conserving the northeastern Atlantic Forest of Brazil, specifically the Pernambuco Endemism Center (PEC), which is the epitome of tropical forest fragmentation worldwide^13^. In the PEC, only 12.2% of the original forest cover remains, existing as small fragments within a matrix dominated by livestock farming and sugarcane cultivation^14^. This fragmented landscape is the result of successive colonization cycles for the extraction of Pau-Brasil (*Paubrasilia schinata*), as well as extensive sugarcane plantations for the production of sugar and fuel alcohol^15^. As a consequence, the biodiversity in the PEC remains significantly endangered, necessitating immediate mitigation measures to ensure the protection of the region’s diverse and endemic flora and fauna^14,15^.

In this context, the overarching objective of this study is to develop a comprehensive framework for prioritizing and connecting forest patches within the PEC of the Brazilian Atlantic Forest. This will be achieved by (i) analyzing the landscape composition, (ii) defining priority forest patches for conservation using an integrative multi-criteria approach, (iii) proposing ecological corridors (ECs) to connect the selected forest patches, and (iv) estimating the financial cost for implementing these ECs through forest restoration. The inclusion of financial cost estimation for implementing ecological corridors through forest restoration introduces a valuable and relatively novel aspect to this study. While most research emphasizes ecological and spatial analyses, this study also addresses the economic feasibility of corridor implementation, which is crucial for the practical application of conservation strategies. This financial assessment enhances the comprehensiveness of the approach, positioning this study as a significant contribution to conservation planning in the Brazilian Atlantic Forest.

## Results

### Landscape ecology analysis

**Fig. 1:**
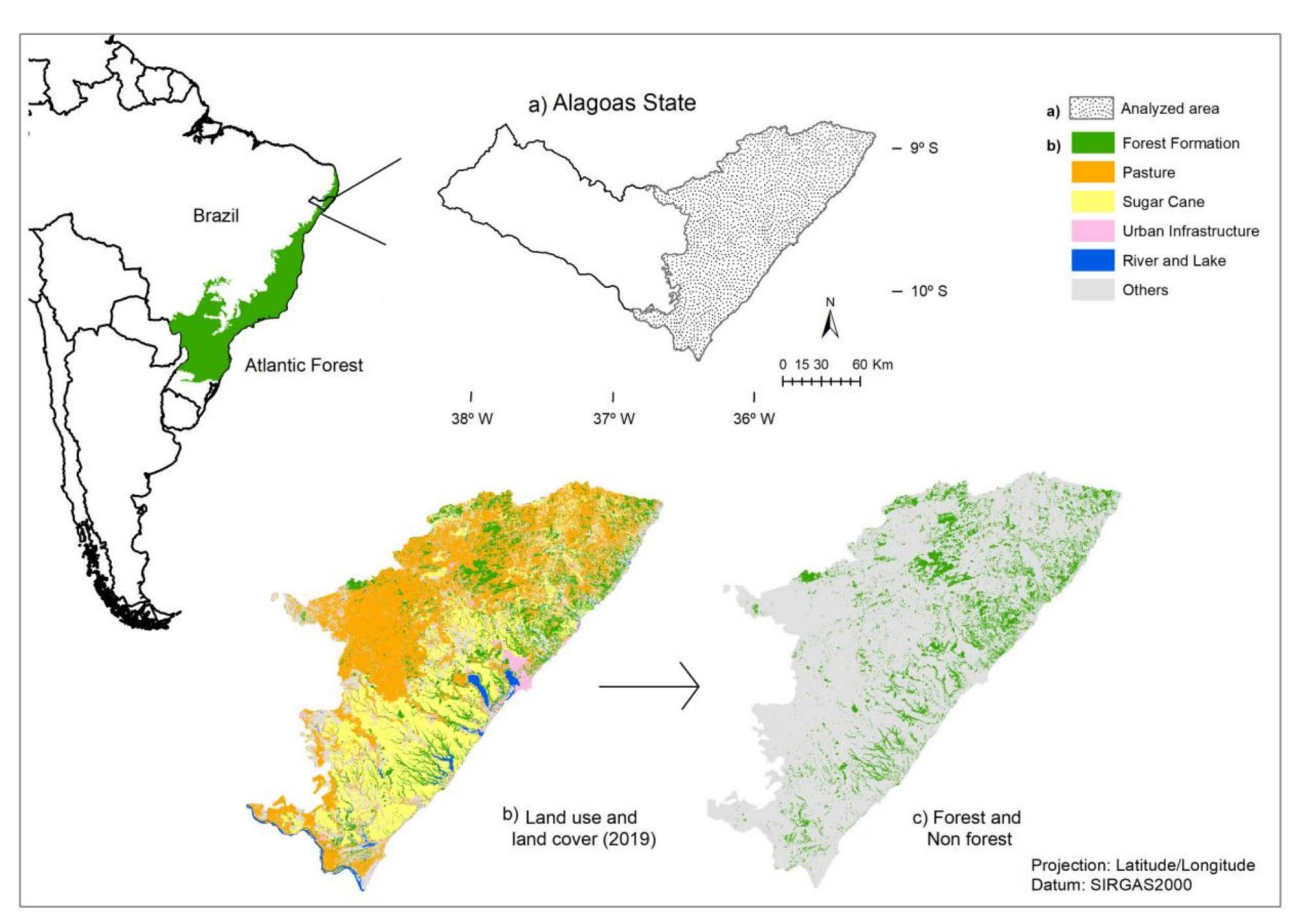
Location map of (a) study area that refers to the Alagoas state - Brazil, specifying (b) land use and land cover and (c) forest and non-forest fragments.

Fourteen different types of land use and land cover were found covering the Atlantic Forest of Alagoas, mostly represented by non-native classes of the landscape, such as Pasture (39%), Sugarcane (25%), Mosaic of Agriculture and Pasture (10%), Other Temporary Crops (8%) and Urban Infrastructure (2%), derived from human presence. Nevertheless, other types of native land cover were found, including Forest (13%), River and Lake (2%), Savanna Formation (1%) and Mangrove, Beach and Dune and other natural vegetation types with less than 1% (Table S1). For enhanced visual representation, just the principal categories of utilization were elucidated in Fig. 1b.

A total of 35,344 forest fragments covering an area of approximately 185,000 hectares were identified. Small fragments (< 10 hectares) dominated the distribution, comprising 94% (33,262 fragments) of the total count. In contrast, medium-sized fragments (10–50 hectares) and large fragments (> 50 hectares) constituted only 3% (1,542 fragments) and 1.5% (540 fragments) of the total, respectively. The smaller fragments exhibited higher Total Edge (TE) and Edge Density (ED) values, indicating a greater susceptibility to edge effects in comparison to the medium and larger fragments. Regarding core metrics, large fragments had the highest Total Core Area (TCA) and Total Core Area Index (TCAI) values, while small fragments showed higher Core Area Density (CAD) values (Table 1). When considering a minimal edge effect buffer of only 50 m, 82% (29,048) of the total fragments in the landscape disappeared, indicating that these fragments lacked a minimum central area and were affected by edge effects throughout their entirety.

**Table 1:** Landscape metrics from the study area, considering size classes of the forest fragments. AREA MN = Mean patch area; CA = class area; CAD = core area density; ED = edge density; NP = number of patches; TCA = total core area; TCAI = total core area index and TE = total edge.

Index	Size classes		
	Small (< 10 ha)	Medium (10–50 ha)	Large (> 50 ha)
CA (ha)	30,855.60	33,531.00	121,269.00
TE (ha)	12,697,100.00	5,445,790.00	10,654,100.00
ED (m.ha^− 1^)	411.50	162.41	87.86
NP (Dimensionless)	33,262	1,542	540
AREA MN (ha)	0.93	21.75	224.57
TCA (ha)	2,101.43	11,567.33	73,730.38
TCAI (%)	1.13	6.23	39.71
CAD (ha)	0.022	0.008	0.002

**Fig. 2:**
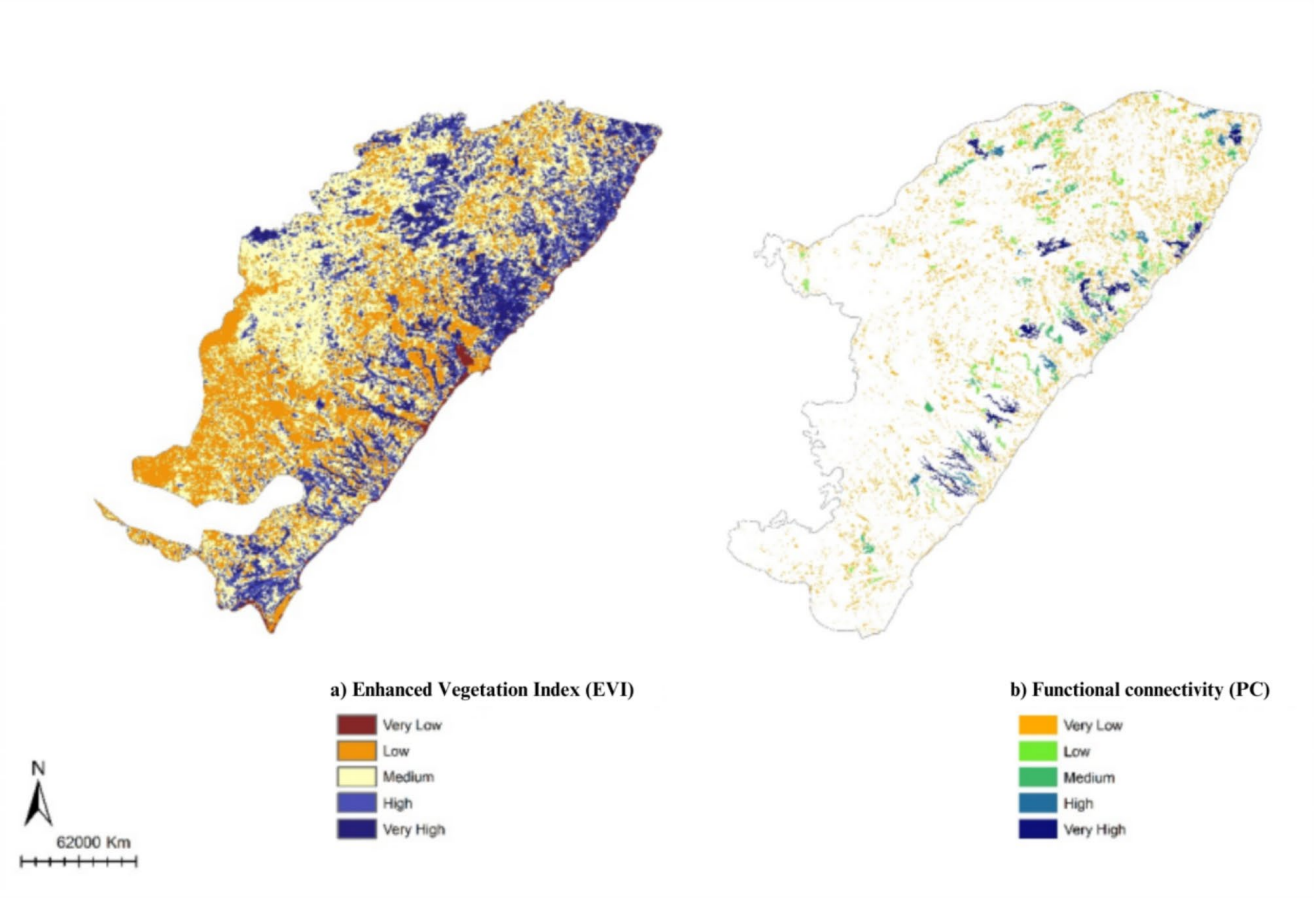
(a) Enhanced Vegetation Index (EVI) and (b) Importance of forest fragments for overall landscape functional connectivity measured by Probability of Connectivity (PC).

The Enhanced Vegetation Index (EVI) revealed that most of the total forest fragments analyzed (14,471 fragments) are in the medium category (EVI – 0.23 to 0.30) 49.07% (or 7,101). The analysis also showed low EVI values (categories very low and low: -1 to 0.22) for 21.02% (or 3,043) of the fragments and a slightly higher amount in the high and very high categories (EVI 0.31 to 1) comprising 29.90% of these fragments (or 4,327) (Fig. 2a). These results indicate that 79% of the vegetation is considered healthy, which this condition varies between 0.2 and 0.8^16^. The best fragments are spatially distributed in the northern quadrant (Fig. 2a). The Probability of Connectivity (PC), however, revealed of the total forest fragments analyzed (39,882 fragments) that the functional connectivity of the studied landscape is quite low, with the vast majority of forest fragments (> 99.44%) presenting very low (PC 0–0,001) PC values represented by the first category analyzed. Subsequent categories showed higher PC values and covered, respectively 150 (low – PC 0,001 − 0,003), 53 (medium – PC 0,003 − 0,034), 13 (high – PC 0,034 − 0,071) and 4 (very high – PC 0,071 − 0,352) forest fragments (Fig. 2b). The largest forest fragments showed the highest PC values.

## Proposals for conserving priority forest fragments

**Fig. 3:**
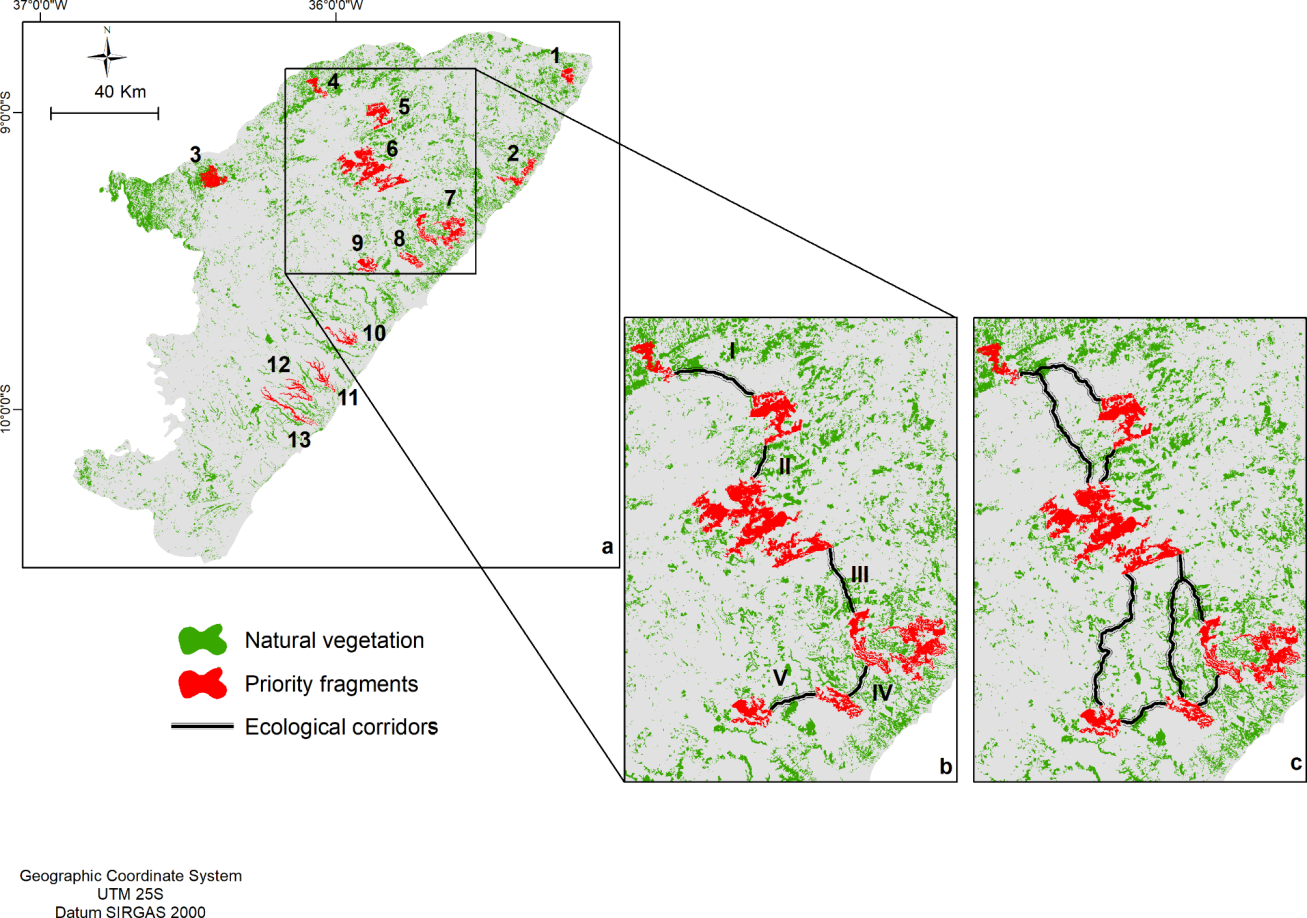
Identification of priority forest fragments (a) ecological corridors using continuous surface (b) and ecological corridors using resistance surface (c) to connect major forest patches in Alagoas, Northeast Brazil.

By employing a multi-criteria approach that integrates EVI and PC, with the addition of fragment size data as a final filter, we were able to identify 13 potentially crucial forest fragments (Fig. 3a). These fragments ranged in size from 1,518.0 to 11,691.2 hectares, collectively covering 42,828.34 hectares or approximately 20% of the total area of all forest remnants in the analyzed landscape (Table S2). While these fragments were generally spatially dispersed across the landscape, there was a noticeable clustering closer to the coast and in the northern quadrant (Fig. 3a).

## Proposals for ecological corridors and forest restoration

The ecological corridors (ECs) aimed to connect priority forest fragments through LCP based using two different approaches, using the Spatial Analyst and the Linkage Mapper tools. The longitudinal direction was predetermined due to the greater heterogeneity of vegetation along the coast than in the latitudinal direction. Both approaches resulted in practically the same paths, but the second was more than 5,000 km away compared to the first, and included three more possible paths to connect some fragments at more than one point (Fig. 3b and c). Therefore, using parsimony, we defined that planning would follow the first approach which identified five corridors linked six forest fragments one by one crossing, spanning 54.1 km and 527.72 ha in total (Fig. 3b; Table 2).

Then the defined corridors were set width of 100 m, varying in length from 6.7 to 16.9 km and covering areas from 65.09 to 164.65 ha (Fig. 3b; Table 2).

These corridors faced land use and cover restrictions, leading to the definition of five classes (Table 2). ‘Natural vegetation’ was predominant, covering 243.61 hectares (46.17%), followed by ‘pasture’ at 122.85 hectares (23.29%). ‘Mosaic agriculture and crops’ and ‘sugarcane’ classes occupied 109.28 hectares (20.72%) and 47.7 hectares (9.05%), respectively. The ‘urban’ class, representing high implementation costs, appeared in only two ECs (I and V), totaling 4.1 hectares (0.77%) (Table 2).

The total area designated for restoration within the ECs was 283.93 hectares, excluding ‘natural vegetation.’ Restoration areas ranged from 25.71 to 115.30 hectares among ECs (Table 2). Restoration costs were calculated based on the opportunity cost of three prevalent classes found in all ECs: ‘pasture,’ ‘sugarcane,’ and ‘Mosaic of Agriculture and Pasture,’ which collectively accounted for 279.83 hectares, constituting over 98% of the total restoration area within ECs. The remaining 2% corresponded to ‘urban’ areas, not requiring expropriation.

The total cost of forest restoration amounted to approximately US$547,683.80, approximately US$1,928.94 per hectare. ‘Mosaic agriculture and crops’ restoration costs exceeded those of ‘sugarcane’ and ‘pasture,’ amounting to US$244,268.42, US$167,218.39, and US$136,721.48, respectively (Table 2). EC II was the corridor with the most financially viable characteristics, exhibiting a relatively small area. Conversely, EC I incurred the highest expenses, due to its higher proportion of pasture and agriculture mosaic areas (Table 2). Permanent Preservation Areas (PPAs) and Legal Reserves (LRs) collectively covered 151.59 hectares within all ECs, corresponding to 62.22% of the natural vegetation in these corridors. PPAs and LRs ranged from 1.01 to 34.05 hectares and from 2.64 to 33.82 hectares within the ECs, respectively (Table S4). Among the ECs, EC II contained the largest contribution of the ‘natural vegetation’ class, amounting to 29.15 hectares. ‘Natural vegetation’ predominated within PPAs and LRs, occupying 124.74 hectares, equivalent to 82.28% of the total area of PPAs and LRs. The classes ‘pasture,’ ‘sugarcane,’ and ‘Mosaic agriculture and crops’ were deemed unsuitable within PPAs and LRs, totaling 26.83 hectares (Table S3).

**Table 2:** Classification of land use and cover (% and ha), length (km) and estimated value for forest restoration (US$) for each ecological corridor established in the study area.

Land use and cover classes	%	ha	US$
*Corridor I – 16.9 km*			
Pasture	35	58.06	60.785,16
Sugarcane	3	4.77	18.089,83
Mosaic agriculture and crops	32	51.99	112.282,13
Urban	0	0.48	
Natural vegetation	30	49.35	
*Corridor II – 6.7 km*			
Pasture	25	16.26	19.010,24
Sugarcane	1	0.50	4.366,90
Mosaic agriculture and crops	14	8.95	19.185,09
Natural vegetation	60	39.20	21.614,07
*Corridor III – 13.9 km*			
Pasture	15	20.35	23.097,79
Sugarcane	11	15.15	51.449,07
Mosaic agriculture and crops	20	26.57	58.732,36
Natural vegetation	54	73.91	
*Corridor IV – 7.1 km*			
Pasture	12	8.59	11.344,85
Sugarcane	15	10.62	36.890,56
Mosaic agriculture and crops	28	10.98	25.890,47
Natural vegetation	57	39.64	
*Corridor V – 9.5 km*			
Pasture	21	19.59	22.338,25
Sugarcane	18	16.66	56.301,91
Mosaic agriculture and crops	12	10.79	25.490,21
Urban	4	3.62	
Natural vegetation	45	41.51	

## Discussion

The landscape analysis reveals extensive fragmentation in the Atlantic Forest, particularly in the PEC biogeographic region^17^. Small fragments (< 10 ha) significantly outnumber medium and large ones (> 10 ha), indicating severe fragmentation. This disrupts landscape structure and adversely affects ecosystem services. However, small fragments can connect larger remnants, facilitating species movement^18^. Due to increased edge effects from habitat loss, smaller fragments inherently support lower species diversity, favoring generalist species and isolating wildlife^19^. Moreover, smaller and medium-sized fragments exhibit lower mean patch area (AREA MN) values, while total edge (TE) and edge density (ED) are higher in smaller fragments. The surrounding matrix, mainly composed of pasture and sugarcane (Table S1), exacerbates edge effects, disproportionately impacting smaller fragments^20^.

Higher total core area (TCA) and Total Core Area Index (TCAI) values are observed in the largest fragments (Table 1). A significant number of fragments (29,048) disappear when considering the core area, as they are too small to withstand edge effects. Core Area Density (CAD) shows higher values in the smallest size classes (Table 1). The reduction in fragment area due to edge effects leads to lower CAD values^20^, compromising habitat quality.

The Enhanced Vegetation Index (EVI) indicates good landscape quality, likely due to the resilience of forest fragments, even smaller ones. However, the Probability of Connectivity (PC) reveals extremely low functional connectivity, primarily limited to large fragments. These metrics assess the importance of habitat patches for both availability and connectivity^21^. Increased distances between fragments correlate with reduced structural connectivity, which limits fauna movement and decreases functional connectivity, particularly for species dependent on forest habitats^22^. Structural connectivity refers to the physical configuration of the landscape, such as fragment distance, while functional connectivity focuses on how species interact with the landscape and their ability to traverse various matrix types^23^. The composition of the matrix between fragments further restricts fauna movement, leading to competition within low-quality patches^24,25^. Our approach, using the PC metric, emphasizes functional connectivity, which is critical for understanding species navigation in fragmented landscapes. However, this method has limitations, as it assumes species movement and matrix permeability that may not fully reflect real-world interactions.

Criteria for prioritizing biodiversity conservation and habitat restoration areas vary but depend on the landscape context^26^. Ecological dynamics at this scale influence processes in restored areas^27^. Metapopulation theory predicts higher colonization in well-connected patches and higher local extinction in small, low-quality ones^28^. Ideally, protected areas should be large, high-quality, and well-connected, as shown in this study. In highly fragmented areas like the PEC, larger fragments should be prioritized, given the severe habitat loss and disconnection^17,29^.

Many studies have already demonstrated the positive influence of the size of fragments on the abundance and diversity of vertebrates in the Atlantic Forest, such as mammals^30^, birds^31^ and reptiles^32^. This study offers an integrative approach to prioritize conservation areas, especially effective when conserving multiple species with varying habitat needs^33^. In heavily impacted landscapes like the PEC, effective conservation tools require ecological attributes like habitat connectivity, vegetation quality, and forest cover, emphasizing the importance of ecological networks such as ecological corridors^14^.

Conservation efforts should prioritize both fragmented and degraded forests while ensuring the protection of non-degraded forests. Priority fragments often lie within pasture and sugarcane matrices, facing threats such as land speculation, hunting, logging, and sugarcane burning. Some of these fragments overlap with conservation units, underscoring the importance of protected areas like the Murici Environmental Protection Area and the Pedra Talhada Biological Reserve. Protecting and expanding these areas is crucial for biodiversity conservation.

The proposed EC system connects six priority fragments through five sections with lower resistance costs, prioritizing larger fragments (Fig. 2b). Our study assesses landscape ecology, identifies priority fragments, defines ECs, and estimates restoration costs. These ECs form a significant corridor crossing the Atlantic Forest of Alagoas state, a novel approach in the PEC region of Brazil, known for its unique and highly threatened biodiversity. Fragment size and landscape connectivity significantly influence species richness and abundance^22,30^, making ECs crucial for mitigating habitat fragmentation effects and conserving biodiversity^30^.

Determining the best path between priority forest fragments aids in estimating wildlife distribution and land use^34^. “Natural vegetation” is the most suitable land cover for EC implementation, dominating all planned ECs and representing the original species habitat. Building ECs requires an understanding of the landscape’s biophysical structure and essential factors contributing to the proposed corridor’s quality and function. Integrating ecological attributes, such as habitat connectivity, vegetation quality, and forest cover, is crucial for long-term species persistence in the face of climate change and human activities^35^.

We utilized two different approaches which has as base LCP modeling in this study to propose corridors based on land use classes, road presence, PPAs, LRs, and the largest fragments in the region, prioritizing variables directing corridor paths^36^. Land use conflicts predominantly result from improper land use, indicating non-compliance with environmental legislation (Brazilian Forest Code - Law nº 12.651/2012) related to PPAs and RLs. Estimated restoration costs, at US$1,928.94 per hectare for ten years, align with Atlantic Forest estimates (US$2,102.83 per hectare; ^37^). EC II stands out for potential implementation due to its high suitability, minimal land use conflicts, including LR areas, and connectivity between the two largest fragments in the area.

Prioritizing forest restoration is crucial for environmental resilience, and rural landowners should explore revenue sources like timber and non-timber forest products or payments for environmental services, recently regulated in Brazil (Law nº 14.119/2021). Additionally, environmental education programs targeting the local population are vital for successful conservation and restoration efforts^38^. For conservation purposes, forest area, fragment size, and connectivity are vital metrics for assessing animal population viability. Our findings align with previous recent studies and raise concerns about the persistence and viability of various species in the evaluated landscape due to the small size and isolation of most fragments^14^. In Alagoas state’s PEC, 41% of fragments are at least 3 km^2^ (300 ha) in size, with an average distance of 3.6 km between the nearest fragments, and about a quarter of forest fragments are isolated within a 6 km radius^14^.

The majority of forest remnants are too small to support long-term viable populations of medium-large or forest-dependent fauna. Small mammals face a particularly challenging situation, as they require a minimum of 13 km^2^ (1,300 ha) for marsupials and 25 km^2^ (2,500 ha) for rodents to ensure demographic and genetic viability^39^. For forest-dependent species or those unable to cross the altered matrix surrounding the fragments, each forest fragment operates as an isolated island, forcing species to venture into sugarcane and pasture to survive in the predominant landscape matrices. The proposed ecological corridor is expected to mitigate landscape fragmentation, potentially restoring connectivity^5^, reducing extinctions^40^, and maintaining biodiversity^41^, based on studies demonstrating the effectiveness of this strategy.

This framework is widely applicable for selecting areas and defining corridors. Establishing forest corridors at reasonable costs safeguards most vertebrate species. These areas involve both small-scale producers and large-scale sugarcane mills, where the implementation costs are minimal relative to the substantial profits generated by these mills^42^. Additionally, these costs are negligible compared to the budgets of environmental agencies^43–45^. Given the size of the corridors, the economic impact on productivity is minimal, including for small-scale landowners. On the other hand, the potential benefits are substantial, as the corridors aid in legal compliance and offer opportunities for compensation programs and financial incentives. Additionally, they provide long-term economic benefits by maintaining ecosystem services and environmental health. Both private and public initiatives should contribute to this effort. Brazil’s commitment to global agreements supports large-scale restoration within existing national policies and promotes carbon credit production in the Atlantic Forest ecosystem, effectively integrating environmental preservation, climate change mitigation, and economic development.

It is important to note that this study is not without limitations, which should be taken into account when interpreting the findings. The absence of a resistance matrix in our connectivity analysis may have resulted in a simplification of habitat complexities, particularly for species with varied habitat requirements. The adoption of a fixed 0.05 displacement capability was intended to facilitate the description of hypotheses regarding migration connectivity or seasonal dispersal. However, the use of alternative displacement values may result in disparate interpretations, as would the application of distinct weighting values to the resistance surface^46,47^. These factors present significant challenges in developing solutions that can be applied to a diverse range of species. Additionally, the economic assessments did not incorporate inflationary adjustments, which could potentially impact the precision of cost projections for corridor implementations. These elements are vital for enhancing future conservation strategies in this highly fragmented landscape.

## Conclusion

This study highlights the extensive fragmentation of the Atlantic Forest, particularly in the PEC biogeographic region, and underscores the importance of large, well-connected forest fragments for biodiversity conservation. In this sense, the proposed ecological corridors offer a practical solution to mitigate fragmentation by increasing connectivity and supporting species movement. Prioritizing the restoration of these corridors, especially in regions dominated by pasture and sugar cane, is critical for maintaining biodiversity and ecosystem services. The results underscore the need for coordinated conservation efforts that integrate ecological connectivity, forest cover, and landscape quality to ensure the long-term viability of species and habitats in the face of ongoing environmental challenges.

### Methods

#### Study area

The northeastern Atlantic Forest of Brazil, also called Pernambuco Endemism Center (PEC) is the global tropical forest experiencing both the highest fragmentation levels and extinction rates^13^. This region is the most devastated within the Atlantic Forest ecosystem, with high levels of endemic species at extinction risk^14^. It represents a conservation hotspot within this ecosystem, which has been considered the hottest of the global priority regions for conservation efforts^48^ and Alagoas state presents the greatest native forest remnants persisting in the landscape^14^. Currently, Alagoas retains about 9.4% of its original Atlantic Forest cover, comprising diverse ecosystems, including ombrophylous and semi-deciduous forests, mangroves, wetlands, and restingas, which is limited to the eastern portion of the state, along the coast (Fig. 1a). The area experiences a tropical monsoon (Am) and tropical dry savanna (As) climate according to the Köppen classification, marked by seasonality in rainfall, minimal temperature variation, and a driest month with less than 60 mm of rainfall^49^. Altitude ranges from 7 to 488 m, with an average temperature of 24.9 °C and average annual precipitation of 114.3 mm, peaking between March and August at around 173.1 mm^49^.

## Landscape ecology analysis

### Step 1. Spatial database and land use and cover map

Data analysis was conducted using ArcGIS 10.5 software with the Universal Transverse Mercator projection (Zone 24 South, datum SIRGAS 2000). Land use and cover data were obtained from MapBiomas collection 5.0 (http://mapbiomas.org) for the year 2019, derived from LandSat images via Google Earth Engine with 30 m resolution. Raster-format classified images were converted to vector shapefiles and clipped to match the study area’s boundaries (Fig. 1b). To conduct the connectivity analysis, an 8 km buffer zone was applied around the state boundaries. This buffer size was chosen because it encompasses the maximum distance a fragment within the study area extends beyond the state’s geopolitical limits. By incorporating this buffer, we ensured that the analysis accurately represents landscape connectivity without introducing artificial fragmentation. It effectively captures the ecological interactions and movements of species that span state borders, ensuring that the buffer captures the full extent of the connectivity in this region.

### Step 2. Landscape ecology analysis

A shapefile of forest fragments was extracted from the land use and cover map by selecting the corresponding polygons (Fig. 1c). The fragments were categorized by size into three classes: (a) small (< 10 ha), (b) medium (10–50 ha), and (c) large (> 50 ha), as adapted from previous research^50^. Landscape analysis was performed using Patch Analyst extension in ArcGIS 10.5^51^ based on raster files of the forest fragments. The selected landscape ecology metrics quantify and describe fundamental physical and spatial characteristics for the analysis of landscape structure and composition (Table 3) and were assessed for each size class to compare conservation levels relative to fragment size. For the Enhanced Vegetation Index (EVI) and Probability of Connectivity (PC) metrics, a classification into five categories was applied: Very Low^1^ to Very High^5^. The classification utilized the Natural Jenk Break method^52^. This method optimally arranges diverse values into different classes by minimizing deviation within classes and maximizing standard deviation among them. It is commonly used for geospatial data clustering^53^.

**Table 3:** Landscape ecology metrics used to quantify the landscape structure in the study area. Sources: ^54^for general landscape metrics, ^55^for the enhanced Vegetation Index (EVI), and ^56^for the probability of Connectivity index (PC).

Metric	Acronym (unit)	Group
Class Area	CA (ha)	Area, density and Edge
Total Edge	TE (ha)	
Edge Density	ED (m.ha^− 1^)	
Number of Patches	NP (Dimensionless)	
Mean Patch Area	AREA MN (ha)	
Total Core Area	TCA (ha)	Core Area
Total Core Area Index	TCAI (Percentage)	
Core Area Density	CAD (ha)	
Enhanced Vegetation Index	EVI (Dimensionless)	Vegetation Health
Probability of Connectivity	PC (Dimensionless)	Functional Connectivity

Class area (CA) was computed for each size class, representing the total area of fragments in that category. Total edge (TE) was determined as the sum of all fragment edges, and edge density (ED) was calculated as TE divided by the total landscape area. The number of patches (NP) indicate the count of fragments for each land use and cover type, reflecting landscape fragmentation. Mean patch area (AREA MN) represented the average fragment size, while total core area (TCA) was the sum of core areas within fragments. Core areas were defined as regions at least 50 m from fragment edges since this is the distance in which the vegetation dynamics and structure are more affected by edge effects in the PEC^57,58^. The total core area Index (TCAI) summed core areas and divided by the total landscape area, while core area density (CAD) was calculated by summing disjunct core areas within each fragment of the corresponding patch type and dividing by the total landscape area.

EVI was developed to enhance the sensitivity to structural variations in the canopy, particularly in areas with dense vegetation, by reducing atmospheric and soil influences^55^. We utilized 16-day composite data (from 25/11/2019 to 10/12/2019) from the Moderate Resolution Imaging Spectroradiometer (MODIS) sensor aboard the TERRA satellite, which has a spatial resolution of 250 m (https://terra.nasa.gov/data/modis-data). For our analysis, we employed four spectral bands from Landsat-8, namely, blue (459–479 nm), red (620–670 nm), near-infrared (841–876 nm), and short-wave infrared (2105–2155 nm).

The connectivity metric used was Probability of Connectivity (PC), quantifying the likelihood of two dispersers placed randomly within the landscape falling into interconnected habitat areas based on habitat patches and their connections, and representing functional connectivity^56^. This metric employed the delta mode, which calculates connectivity by temporarily removing a local graphic element (such as a node or link representing forest fragments or ecological corridors, respectively). The value is determined both before and after removing the graphic element, resulting in an index^59^. PC is considered a valuable metric for objective landscape conservation planning and land-use change analysis^56^. It ranges from 0 to 1, with higher values indicating improved connectivity and assigns a value to each fragment based on its contribution to overall regional connectivity^56^. It can be calculated at different scales, reflecting the maximum displacement distance of the studied organisms. In this study, we used a spatial scale of 300 m based on the average maximum displacement distances of small terrestrial mammal species in the Pernambuco Endemism Center (see Table 13.1 in Feijó et al. 2023; Table S5). This scale is appropriate because multiple studies have shown that small mammals in the Atlantic Forest generally do not move more than 300 m, exhibiting limited movement ranges^60–62^. A focus on small terrestrial mammals, which exhibit comparable ecological characteristics such as movement patterns, habitat use, and energy requirements, facilitates a more uniform analysis. This approach serves to minimize the potential for ecological variability to arise from the inclusion of species with differing traits, such as large herbivores or arboreal mammals. By focusing on this group, we can reduce the potential for bias associated with the lack of species-specific data. This guarantees that our findings more accurately reflect the needs and behavior of species with comparable ecological requirements. The matrix’s influence on species movement was disregarded, considering only displacement capacity. The analysis defined forest fragments as habitat and other landscape elements as non-habitat, assuming a homogeneous landscape with equal probabilities of movement between habitat elements (*p* = 0.05). PC calculations were performed using Graphab 2.6 software, specialized in modeling ecological networks^63^.

### Priority fragments for conservation

#### Step 1. Database

The employed spatial database consisted of: (a) forest fragments; (b) Enhanced Vegetation Index (EVI); and (c) Probability of Connectivity (PC).

### Step 2. Spatial data procedures

After characterizing the study area using landscape metrics, Probability of Connectivity (PC), and Enhanced Vegetation Index (EVI), a Weighted Sum model was applied. To focus on larger, more ecologically significant forest fragments, a final filter was used to exclude fragments smaller than 50 ha, as smaller fragments tend to support lower functional biodiversity^38,64^. This entire process was conducted within the ArcGIS 10.5 software.

The Weighted Sum model, a multi-criteria analysis, was employed in conjunction with the Fuzzy Logic model based on expert knowledge and discussions^65^. Fragment relevance classes were created by categorizing fragments into five groups: Very Low^1^ to Very High^5^ relevance. The Natural Jenks Break classification method was used due to the non-normal and non-uniform nature of the data. Fuzzy logic was applied to attributes represented as linear functions [y = f(x)], with values ranging from zero to one to standardize them^66^. The Weighted Sum model was then used to combine EVI with functional connectivity. Both EVI and functional connectivity were assigned equal weights of 0.50 in the Weighted Sum model. This decision was based on the premise that both variables are equally important in determining priority fragments, as both enhance conservation efforts and together can better prioritize areas that have the greatest potential to support species and ecosystem services over the long term^67,68^.

Ultimately, fragments deemed important for conservation were identified by considering the last two relevance classes: High^4^ and Very High^5^.

### Ecological corridors (ECs)

#### Step 1. Database

The spatial database used comprised of: (a) predefined forest fragments (priority fragments for conservation, as above); (b) origin polygon; (c) destination polygon; (d) Permanent Preservation Areas (PPAs) and Legal Reserves (LRs), where restoration is mandatory; (e) land use and cover; and (f) paved roads. The predefined forest fragments file was used in the analysis to function as origin and destination polygons, for each EC analyzed.

The “forest fragments predefined”, PPAs, LRs, “land use and cover” and “paved roads” files for the study area (in vector format), were converted to raster format (function Polygon to raster). PPAs and LRs were obtained from the Rural Environmental Register (Cadastro Ambiental Rural - CAR) through its electronic system (https://www.car.gov.br/#/) created through Brazilian Law nº 7.830/2012. Paved roads data was obtained through the Environmental Institute (Instituto do Meio Ambiente) of the state of Alagoas (https://www.ima.al.gov.br/).

### Step 2. Least-cost path analysis

In order to ascertain optimal strategies for implementing ECs, two analytical approaches based on LCP methodologies were employed to identify the least costly path between two forest fragments. Both analyses were conducted in ArcGIS 10.5 software using distinct tools, Spatial Analyst and Linkage Mapper. The first approach is more generic and broadly applicable for LCP analysis, while the second is specifically designed to optimize ecological connectivity, taking into account detailed aspects of landscape ecology and species movement needs^69^.

Here, selected factors that may influence the passage of EC in the landscape have been selected as paved roads, land use and land cover and PPAs and RLs. The cost of each cell is represented by weights, based on some factor, or combination of factors, that affect the passage of EC through the area. The definition of weights was adapted from Louzada and collaborators^70^ and used equally for both tools, assigned on a scale of 1 to 9 according to the different classes of each factor (Table S4). Weights reflect the ecological importance and management feasibility of landscape elements for maintaining connectivity. Lower weights indicate higher connectivity priority, as in the case of Permanent Preservation Areas and Legal Reserves, which facilitate ecological corridors at lower implementation costs. Higher weights indicate significant barriers, such as paved roads that disrupt animal movement. A detailed rationale for these weights is provided in Table S4.

In the Spatial Analyst tool, from the normalized comparison matrices, we calculated the final statistical weights for the total cost matrix generated by Weighted Overlay tool, adding the weights of each layer sum equal to 100% (Table S4). From the total cost matrix image, the Cost Distance and Cost Direction images were generated (Backlink) and formed the basis for constructing the least cost path. We used the best single path (Best Single) it performed from fragment to the nearest fragment, successively.

Using the Linkage Mapper tool, an integrated resistance surface was generated employing identical weighting methods for all resistance factors (layers). Following the determination of the resistance surface and effective range, the Linkage Mapper toolbox software was utilized to delineate the LCPs. In Linkage Mapper 2.0, a GIS toolkit developed by McRae and Shah^71^, the Linkage Pathway tool utilizes the Minimum Cumulative Resistance model, weighted by Euclidean distance, to identify LCPs^10^. Initially, centroids of forest fragments were extracted and designated as nodes. Subsequently, these nodes along with the resistance surface were inputted into the Linkage Pathway tool. This tool computed a cost-weighted distance surface by evaluating the cost-weighted distance (CWD) weighted by Euclidean distance for each cell from neighboring nodes. This facilitated the determination of the minimum cost-weighted distance between nodes and consequently enabled calculation of LCPs connecting the nodes, representing potential corridors.

The LCP correspond to areas with a higher probability of movement. While LCP analysis assumes that individuals possess perfect knowledge of the landscape, allowing them to follow an optimal path, its biological realism can be questioned. Nonetheless, this method is valuable when there is limited input data available^72^ and is easily accessible to landscape planners. In each path generated for the ECs, a buffer with a width of 100 m was created. This width was defined to be functional for most of the biodiversity present in the region and was based on studies of the implementation of corridors considering groups of species and sensitivity of the species to habitat disturbances that evidence the 100 m supporting the movement of small amphibians and mammals, birds, including some sensitive species, and middle mammals^73–75^. Furthermore, this width is already used by landowners who implemented corridors in the region of PEC.

### Step 3. Land use conflicts in the ecological corridors

Using the results of the Spatial Analyst tool, an analysis of land use conflicts was carried out within each EC based on the spatial overlap analysis. The results were vectorized and each path was analyzed individually as to its length and intersection with the information on land use and land cover. Since the urban class was practically insignificant in the ECs, we chose to focus the analysis of land use conflicts in relation to PPAs and RLs, considering land use conflicts in PPAs and LRs, mostly are due to improper use of the land, evidencing non-compliance with current environmental legislation (Forest Code - Brazilian Law nº 12.651/2012). In the analysis, the percentage of occurrence of each class identified was evaluated using the land use and land cover map, including the PPAs and LRs layers.

### Forest restoration

The forest restoration proposal aims to enhance connectivity among priority forest fragments identified in this study by restoring ecological corridors (ECs) identified here (Fig. 2b). The restoration area corresponds to the length of each EC multiplied by a fixed width of 100 m.

To estimate the restoration costs in this scenario, we considered both land opportunity costs and implementation costs. Land opportunity costs were calculated based on the annual accumulated rental prices for sugarcane and cattle ranching, which are the dominant land uses in the region. We considered a 10-year time-frame, which is a reasonable period for establishing restored forests^76^. Sugarcane values were based on production income per hectare while livestock income was estimated as pasture rent per adjusted head per hectare. The annual rental price for sugarcane standard was estimated an average at US$321.38/hectare/year (US$246.57 to US$404.62) and for pasture at US$99.94/hectare/year (US$85.64 to US$111.34), based on data from local producers in Alagoas through interviews, and later the data were confirmed through websites widely used for this type of consultation in agribusiness in Brazil (August 2021 to August 2022 reference − ^77,78^). For the ‘Mosaic of Agriculture and Pasture’ land use and land cover we used the average of both values mentioned above, that is, US$210.66/hectare/year (US$166.10 to US$257.98). Land rental costs have not been adjusted for inflation. For the implementation costs, we considered the costs for establishing and maintaining restoration plantations (i.e., mixed plantations of native trees that are weeded up to three years after planting), as the low resilience of the study sites would not allow the use of passive restoration. We considered the cost of planting trees to be US$2.760/hectare, based on a mean of values estimated for environmental favorable and unfavorable conditions in the Atlantic Forest of Brazil^79^. We calculate the cumulative opportunity cost of land by multiplying the aforementioned average annual costs by the age of the restored forests. Total restoration cost was calculated by summing implementation and land opportunity costs multiplied by the area of the ECs (e.g., total cost for 10-year-old restoration plantation in sugarcane = US$2.760.ha^− 1^ + US$321.38.ha^− 1^.year^− 1^ × 10 years × EC area).

## Electronic supplementary material

Below is the link to the electronic supplementary material.

Supplementary Material 1

## Data Availability

The datasets generated and/or analyzed during the current study are available from the corresponding author on reasonable request.
