# Supplementary material for: Priority areas and implementation of ecological corridor through forest restoration to safeguard biodiversity
Source: Sci Rep. 2024 Dec 28;14:30837. doi: 10.1038/s41598-024-81483-y (PMC11681200; doi:10.1038/s41598-024-81483-y)
Supplement: Supplementary file 1 — Supplementary Material 1 [file 41598_2024_81483_MOESM1_ESM.pdf]

Supplementary Material

Priority areas and implementation of ecological corridor through forest restoration to safeguard biodiversity

Mayara Guimarães Beltrão[1,*], Camila Francisco Gonçalves[2,6], Pedro H. S. Brancalion[3], Ana Paula Carmignotto[4], Luis Fábio Silveira[1], Pedro Manoel Galetti Jr.[5], Mauro Galetti[6]

[1]Museu de Zoologia da Universidade de São Paulo, São Paulo, SP, Brazil
[2]Programa de Pós-Graduação em Ecologia e Recursos Naturais, Universidade Federal de São Carlos, São Carlos, SP, Brazil
[3]Departamento de Ciências Florestais, Escola Superior de Agricultura Luiz de Queiroz, Piracicaba, SP, Brazil
[4]Departamento de Biologia, Universidade Federal de São Carlos, Sorocaba, SP, Brazil
[5]Departamento de Genética e Evolução, Universidade Federal de São Carlos, São Carlos, SP, Brazil
[6]Departamento de Biodiversidade, Universidade Estadual Paulista, Rio Claro, SP, Brazil
*mayarabeltrao@gmail.com / +55 83 99912-2272

Table S1 Land use and cover types recorded in the study area.

**Land use and land cover**		**Hectare**	**Percentage (%)**
Pasture		565828,88	39
Sugarcane		366367,59	25
Forest		185747,39	13
Urban Infrastructure		24464,35	2
River, Lake and Ocean		24193,82	2
Others	Mosaic of Agriculture and Pasture	145735,43	10
	Other Temporary Crops	123461,51	8
	Savanna Formation	19514,53	1
	Beach and Dune	4730,05	0
	Mangrove	4211,22	0
	Other Non-Forest Native Formation	2609,05	0
	Other Non Vegetated Area	575,68	0
	Aquaculture	35,34	0
	Salt Flat	60,07	0

Table S2 Identification (Id), size (ha) and location (decimal degrees) of the thirteen priority

forest fragments for the study area. Categories of conservation units according to Brazilian legislation: APA = Environmental Protection Area (in Portuguese, Área de Proteção Ambiental); APP = Permanent Preservation Area (in Portuguese, Área de Preservação Permanente); ESEC = Ecological Station (in Portuguese, Estação Ecológica); REBIO = Biological Reserve (in Portuguese, Reserva Biológica); RL = Legal Reserve (in Portuguese, Reserva Legal); RPPN = Private Natural Heritage Reserve (in Portuguese, Reserva Particular do Patrimônio Natural).

**Fragment Id**	**Fragment size**	**Geographic coordinates**		**Fragment name**
		Longitude	Latitude	
1	1,636.1	-35.216559°	-8.894179°	RPPN Eco Fazenda Morim/RL locais
2	2,418.7	-35.365161°	-9.221726°	RL/APP private
3	4,350.5	-36.423631°	-9.236324°	REBIO Pedra Talhada
4	1,543.7	-36.069309°	-8.925022°	RL/APP Serra Grande
5	3,784.8	-35.849775°	-9.013029°	RL/APP Coimbra
6	11,691.2	-35.896117°	-9.186831°	APA/ESEC Murici
7	6,599.6	-35.612793°	-9.424574°	RPPN Carlos Lyra/RPPN Placas
8	1,518.0	-35.743227°	-9.509216°	RL/APP private
9	1,943.4	-35.902415°	-9.527661°	RPPN Mata do Cedro
10	1,806.3	-35.966321°	-9.772597°	RL/APP private
11	1,765.4	-36.054924°	-9.907802°	RL/APP private
12	1,843.1	-36.123073°	-9.944656°	RL/APP private
13	1,927.3	-36.158540°	-10.017720°	RL/APP private

Table S3 Land use and cover conflicts in Permanent Preservation Areas (PPAs) and Legal Reserves (LRs) (% and ha) for each ecological corridor established between priority fragments in the study area.

**Land use and cover classes**	**PPA**		**LR**	
	**%**	**ha**	**%**	**ha**
Corridor I				
Pasture	7.16	2.44	23.10	0.61
Sugarcane	0.64	0.22		
Mosaic agriculture and crops	14.33	4.88	65.90	1.74
Natural vegetation	77.85	26.51	10.98	0.29
Total		34.05		2.64
Corridor II				
Pasture			4.07	1.33
Mosaic agriculture and crops			6.53	2.13
Natural vegetation			89.38	29.15
Total				32.61

Corridor III				
Pasture	18.03	0.46	7.33	2.38
Sugarcane			1.10	0.36
Mosaic agriculture and crops	12.15	0.31	6.16	2.00
Natural vegetation	69.80	1.78	85.39	27.72
Total		2.55		32..46
Corridor IV				
Pasture	21.78	0.22	4.08	1.38
Sugarcane	13.86	0.14	1.24	0.42
Mosaic agriculture and crops	42.57	0.43	10.79	3.65
Natural vegetation	21.79	0.22	83.88	28.37
Total		1.01		33.82
Corridor V				
Pasture	100	1.52	1.82	0.20
Mosaic agriculture and crops			0.27	0.03
Natural vegetation			97.89	10.70
Total		1.52		10.93

Table S4 Values used for Least Cost Path analysis. The weights for each class (1-9 scale) and for each layer (%) were used in the Spatial Analyst tool, which considers different types of costs over a continuous surface. The same class weight values were used in the Linkage Mapper tool to create the resistance surface.

**Layer**	**Layer weight**	**Class**	**Class weight**	**Justification**
Permanent Preservation Area (PPA) and Legal Reserves (LR)	15%	No	9	Appropriate for corridors and low implementation cost - Sparovek et al. (2012)
		Yes	1	
Predefined Forest Fragments	20%	No	9	Important for the maintenance of local and regional species diversity - Pardini et al. (2005); Step-stones - Garcez da Rocha et al. (2021)
		Yes	1	
Paved Roads	20%	No	1	High negative impacts - barrier, disturbance - Galantino et al. (2022); Ascensão et al.
		Yes	9	

				(2017)
Land use and cover	45%	Natural Vegetation	1	Urban areas constitute barriers for the majority of species - McKinney (2002); Fauna moves preferentially through natural areas and areas with less anthropogenic disturbances - Umetsu and Pardini (2007)
		Pasture	5	
		Sugarcane	5	
		Urban infrastructure	9	
		Mosaic agriculture and crops	3	
		River	5	
		Others	3	

Table S5 A comprehensive list of all small terrestrial mammal species to the Pernambuco Endemism Center in the state of Alagoas, Brazil. The list has been adapted from the original publication by Feijó et al. (2023).

Species
**DIDELPHIMORPIA**
**Didelphidae**
Caluromys philander (Linnaeus, 1758)
Marmosa murina (Linnaeus, 1758)
Marmosa demerarae (Thomas, 1905)
Monodelphis americana (Müller, 1776)
Monodelphis domestica (Wagner, 1842)
Cryptonanus agricolai (Moojen, 1943)
Gracilinanus agilis (Burmeister, 1854)
**LAGOMORPHA**
**Leporidae**
Sylvilagus brasiliensis (Linnaeus, 1758) *
**RODENTIA**
**Caviidae**
Cavia aperea Erxleben, 1777
Galea spixii (Wagler, 1831)
Kerodon rupestris (Wied- Neuwied, 1820)

Echimyidae

Phyllomys sp.

Cricetidae

Akodon cursor (Winge, 1887)

Necromys lasiurus (Lund, 1840)

Oxymycterus dasytrichus (Schinz, 1821)

Cerradomys langguthi Percequillo, Hingst-Zaher & Bonvicino, 2008

Holochilus oxe Prado, Knowles & Percequillo, 2021

Hylaeamys oniscus (Thomas, 1904) *

*Nectomys squamipes (*Brants, 1827)

Oecomys catherinae Thomas, 1909

Oligoryzomys nigripes (Olfers, 1818)

Oligoryzomys stramineus Bonvicino & Weksler, 1998

Oligoryzomys mattogrossae (J. A. Allen, 1916)

Rhipidomys mastacalis (Lund, 1840)

REFERENCES

Ascensão, F., Lucas, P. S., Costa, A., & Bager, A. (2017). The effect of roads on edge permeability and movement patterns for small mammals: A case study with Montane Akodont. Landscape Ecology, 32(4), 781–793.

Galantino, A., Ascensão, F., & Mira, A. (2022). Effects of road verge management on small mammal movement in Mediterranean landscapes. Journal of Applied Ecology, 59(3), 674–685.

Garcez da Rocha, É., Brigatti, E., Niebuhr, B. B. S., Ribeiro, M. C., & Vieira, M. V. (2021). Dispersal movement through fragmented landscapes: The role of stepping stones and perceptual range. Landscape Ecology, 36, 3249–3267.

McKinney, M. L. (2002). Urbanization, biodiversity, and conservation. BioScience, 52(10), 883–890.

Pardini, R., Souza, S. M., Braga-Neto, R., & Metzger, J. P. (2005). The role of forest structure, fragmentation, and local scale in shaping small mammal communities in the Brazilian Atlantic Forest. Biological Conservation, 124(2), 253–266.

Sparovek, G., Berndes, G., Klug, I. L. F., & Barretto, A. G. O. P. (2012). Brazilian agriculture and environmental legislation: Status and future challenges. Environmental Science & Technology, 46(10), 5284–5293.

Umetsu, F., & Pardini, R. (2007). Small mammals in a mosaic of forest remnants and anthropogenic habitats: Evaluating matrix quality in an Atlantic Forest landscape. Landscape Ecology, 22(4), 517–530.
